# Evidence-based definition of hypoprolactinemia in European men aged 40–86 years: the European male ageing study

**DOI:** 10.1007/s11154-024-09890-0

**Published:** 2024-06-03

**Authors:** Thang S. Han, Leen Antonio, György Bartfai, Terence W. O’Neill, Margus Punab, Giulia Rastrelli, Mario Maggi, Jolanta Słowikowska-Hilczer, Jos Tournoy, Dirk Vanderschueren, Michael E. J. Lean, Ilpo T. Huhtaniemi, Frederick C. W. Wu, Ana I. Castro, Marcos C. Carreira, Felipe F. Casanueva

**Affiliations:** 1 Institute of Cardiovascular Research, Royal Holloway University of London, Egham, TW20 0EX UK; 2 Department of Endocrinology, Ashford and St Peter’s NHS Foundation Trust, Chertsey, GU9 0PZ UK; 3 Department of Clinical and Experimental Medicine, Laboratory of Clinical and Experimental Endocrinology, KU Leuven, Leuven, Belgium; 4 Department of Obstetrics, Gynaecology and Andrology, Albert Szent-Gyorgy Medical University, Szeged, Hungary; 5 Centre for Epidemiology Versus Arthritis, Manchester Biomedical Research Centre, The University of Manchester & NIHR, Manchester University NHS Foundation Trust, Manchester, UK; 6 Andrology Clinic, Tartu University Hospital, Institute of Clinical Medicine, University of Tartu, Tartu, Estonia; 7 Andrology, Women’s Endocrinology and Gender Incongruence Unit - Careggi Teaching Hospital, Department of Experimental and Clinical Biomedical Sciences “Mario Serio”, University of Florence, Florence, Italy; 8 Endocrinology Unit - Careggi Teaching Hospital, Department of Experimental and Clinical Biomedical Sciences “Mario Serio”, University of Florence, Florence, Italy; 9 Department of Andrology and Reproductive Endocrinology, Medical University of Łódź, Łódź, Poland; 10 Department of Geriatrics, University Hospitals Leuven, Leuven, Belgium; 11 Department of Public Health and Primary Care, KU Leuven, Leuven, Belgium; 12 Department of Human Nutrition, University of Glasgow, Glasgow, UK; 13 Institute of Reproductive and Developmental, Department of Metabolism, Digestion and Reproduction, Imperial College London, Hammersmith Campus, London, UK; 14 Division of Endocrinology, Diabetes & Gastroenterology, School of Medical Sciences, Faculty of Biology, Medicine and Health, University of Manchester, Manchester, UK; 15 Department of Medicine, CIBER de Fisiopatología Obesidad y Nutricion, Instituto Salud Carlos III, , Santiago de Compostela University, Complejo Hospitalario Universitario de Santiago (IDIS), Santiago de Compostela, CB06/03 Spain

**Keywords:** Hypoprolactinemia, Pituitary function, Reference values, Type 2 diabetes, Health outcomes

## Abstract

Empirical evidence for a low normal or reference interval for serum prolactin (PRL) is lacking for men, while the implications of very low PRL levels for human health have never been studied. A clinical state of “PRL deficiency” has not been defined except in relation to lactation. Using data from the European Male Ageing Study (EMAS), we analyzed the distribution of PRL in 3,369 community-dwelling European men, aged 40–80 years at phase-1 and free from acute illnesses. In total, 2,948 and 2,644 PRL samples were collected during phase-1 and phase-2 (3 to 5.7 years later). All samples were analysed in the same centre with the same assay. After excluding individuals with known pituitary diseases, PRL ≥ 35 ng/ml, and PRL-altering drugs including antipsychotic agents, selective serotonin reuptake inhibitors, or dopamine agonists, 5,086 data points (2,845 in phase-1 and 2,241 in phase-2) were available for analysis. The results showed that PRL declined minimally with age (slope = -0.02) and did not correlate with BMI. The positively skewed PRL distribution was log-transformed to a symmetrical distribution (skewness reduced from 13.3 to 0.015). Using two-sigma empirical rule (2[]SD about the mean), a threshold at 2.5% of the lower end of the distribution was shown to correspond to a PRL value of 2.98ng/ml. With reference to individuals with PRL levels of 5-34.9 ng/ml (event rate = 6.3%), the adjusted risk of developing type 2 diabetes increased progressively in those with PRL levels of 3-4.9 ng/ml: event rate = 9.3%, OR (95% CI) 1.59 (0.93–2.71), and more so with PRL levels of 0.3–2.9 ng/ml: event rate = 22.7%, OR 5.45 (1.78–16.62). There was also an increasing trend in prediabetes and diabetes based on fasting blood glucose levels was observed with lower categories of PRL. However, PRL levels were not associated with cancer, cardiovascular diseases, depressive symptoms or mortality. Our findings suggest that a PRL level below 3 ng/ml (64 mlU/l) significantly identifies European men with a clinically-important outcome (of type 2 diabetes), offering a lower reference-value for research and clinical practice.

## Introduction

Hyperprolactinemia, induced by pituitary adenomas or treatment with estrogens or psychotropic and other drugs, has been extensively studied [1, 2]. Prolactin (PRL), with its emphasis on hyperprolactinemia, is the only pituitary hormone for which lower limits of normal and reference intervals are not established. Its deficiency is only clinically apparent in post-partum women who suffer failure of lactation with PRL ranging between undetectable levels [3] and up to 7.6–12.4 ng/ml [4, 5]. Hypoprolactinemia appears to be associated with cardiometabolic risk in women [4, 6], but no studies have been performed which can establish whether a normal lower limit of PRL can be defined in men.

Like other pituitary hormones, PRL exerts its physiological action within a certain range, but reference intervals are variably reported in existing literature and clinical practice [1, 2]. The production of PRL differs between sexes and possibly varies with age and adiposity [7, 8]. High [1, 2, 9] or low [10, 11] serum prolactin may result from disease processes, surgery and drugs. Physiological secretion of PRL is under tonic inhibitory control by hypothalamic dopamine. Any dopamine blockade, for example from neuroleptic drug treatment [1, 2], will increase PRL levels and any dopamine receptor agonist will reduce it [1, 2]. Although there is evidence and consensus on the definition of hyperprolactinemia [1, 2], there is a lack of empirical evidence for the definition of hypoprolactinemia. The lower limits of PRL quoted by manufacturers of commercial assays are arbitrarily-determined, varying between < 1.8 ng/ml (38 mIU/l) and < 5 ng/ml (106 mIU/l) [12–16]. Some studies completely omit any lower limit [17, 18].

To address this knowledge gap, we have accessed the extensive European Male Ageing Study (EMAS) data from 3,369 middle-aged and older community-dwelling European men free from acute diseases, to establish a lower biochemical threshold of serum PRL level which may be associated with adverse health outcomes.

## Methods

### Participants and study design

The study design and recruitment strategy for EMAS have been described in detail previously [19, 20]. Briefly, at phase-1 of the study, an age-stratified sample of 3,369 men aged 40–80 (mean ± SD: 60 ± 11) years was recruited randomly from population registers in eight European centres (Florence, Italy; Leuven, Belgium; Malmö, Sweden; Manchester, UK; Santiago de Compostela, Spain, Łódź, Poland; Szeged, Hungary; Tartu, Estonia). Participants were assessed at phase-1 (2003–05) and a median of 4.3 (range 3 to 5.7) years later at phase-2 survey (2008–10) [19, 20]. Ethical approval for the study was obtained in accordance with local requirements in each center. All participants provided written informed consent.

The medical conditions and drug history were documented by questionnaires. Type 2 diabetes was identified for those who were recorded with a history of diabetes and treated with an oral antihyperglycemic agent (alpha-glucosidase inhibitor, biguanide, glinide, peroxisome proliferator-activated receptor gamma (PPAR-γ) agonist or sulphonylurea), with or without insulin therapy. Those who were treated with insulin alone were considered to have type 1 diabetes.

### Incidence of type 2 diabetes

The incidence of type 2 diabetes was identified as those who did not have diabetes in phase-1, but in phase-2 reported to have type 2 diabetes or found to have a fasting blood glucose level ≥ 7 mmol/l.

### Measurements

Measurements were recorded for age (years), height (m), weight (kg), waist circumference (cm), PRL in ng/ml (conversion factor: ng/ml = mIU/l [] 0.047), lifestyle factors including tobacco and alcohol and medical and drug history including diabetes, cardiovascular or cerebrovascular disorders, cancer or neoplasia, psychiatric disorders, dopamine agonists, antipsychotic agents and the antidepressants selective serotonin reuptake inhibitors (SSRIs). For the purpose of this analysis, smoking status was classified as never, former or current; and alcohol consumption as non-drinkers, infrequent alcohol drinkers (1–4 days/week) or frequent alcohol drinkers (≥ 5 days/week). Body weight and height were measured by electronic scales (SECA, Hamburg, Germany) and stadiometer (Holtain, Crymych, UK) and BMI was calculated as weight/height^2^ (kg/m^2^). Waist circumference was measured at the level between the lowest ribs and anterior suprailiac crest using non-stretchable tape measure [21]. Depressive symptoms were assessed by Beck depression inventory (BDI).

### Hormone measurements and biochemistry

A single fasting morning (before 10:00 am) venous blood sample was obtained in stress-free environments and processed serum stored at − 80 °C [22]. Measurement of PRL was performed using a chemiluminescence immunoassays (Modular Roche, Milan, Italy). All samples were analysed at the same time and in one center. The coefficient of variation of this method was 0.8–1.7% for repeatability and 1.4–1.8% for intermediate precision, with lower limit of quantification (LOQ) of 0.047 ng/ml (1 mIU/l) [23]. There were 2,948 PRL samples collected in phase-1, and 2,644 samples in phase-2 (3 to 5.7 years after phase-1) of the study. Fasting blood glucose and insulin levels, as well as total testosterone (TT) and estradiol (E2) were also obtained. Measurement of TT was made by liquid chromatography-tandem mass spectrometry [24]. The LOQ was 0.25 nmol/l and coefficients of variation were < 10% within and between runs. Measurement of E2 was performed by gas chromatography-tandem mass spectrometry [25]. The LOQ for E2 was 7.34 pmol/l and coefficients of variation were < 5% within and between runs. Free T was calculated from TT levels, sex hormone binding protein, and albumin concentrations [26]. Blood glucose levels were categorised according to international criteria (normal < 5.6 mmol/l, prediabetes = 5.6–6.9 mmol/l, and diabetes ≥ 7 mmol/l). Homeostatic model assessment for insulin resistance (HOMA-IR) was calculated from fasting blood glucose and insulin [27].

### Exclusion criteria

After excluding pituitary and confounding diseases, severe hyperprolactinemia (PRL ≥35 ng/ml) [28], and drugs that could alter circulating PRL levels including antipsychotic and SSRI anti-depressive agents, there were 5,086 data points (2,845 in phase-1 and 2,241 in phase-2) available for analysis.

### Statistical analysis

Data analyses were conducted using SPSS Statistics for Windows, v.28.0 (IBM Corp., Armonk, NY, USA). The two-sigma empirical rule (approximately two standard deviations (SD) about the mean) was used to determine the group of individuals with a PRL level within the lowest 2.5% amongst all participants (equation: mean – 1.96[]SD). Such individuals are considered to have hypoprolactinemia. Fisher exact test was used to explore the relationship between groups of PRL at different cut-offs with clinical outcomes. If there was evidence of a significant association between variables, logistic regression analysis was performed, and presented as odds ratio (OR) and 95% confidence intervals (CI), both unadjusted and adjusted for age, study centres, smoking status, alcohol consumption, and either BMI or waist circumference.

## Results

Characteristics of patients are summarised in Table 1. At phase-1, the mean (± SD) age was 60 years (± 11.0), BMI 27.8 kg/m^2^ (± 4.1), waist circumference 98.3 cm (± 11.7), PRL levels 8.29 ng/ml (± 3.94) (176 mIU/l ± 84) and glucose 5.65 mmol/l (± 1.45). In phase-2, these individuals’ mean age increased to 63.5 years (± 10.6), with similar BMI (27.9 kg/m^2^ ± 4.3) but waist circumference increased to 99.5 cm (± 11.8), whilst PRL (7.45 ng/ml ± 3.74) and glucose (5.53 mmol/l ± 1.43) did not change significantly. The rates of current smokers (17.1%) and daily alcohol consumptions (16.7%) in phase-1 increased to 21.4% and 23.4%, respectively. Levels of HOMA-IR, TT and FT were significantly (*P* < 0.001) lower in phase-2 than the corresponding values in phase-1. The median E2 value, available in phase-1 only, was 70.1 (IQR = 56.5–86.9), and median values for E2/TT and E2/FT were 4.4 (3.4–5.5) and 0.24 (0.19–0.31), respectively.

**Table 1:** Subject characteristics in phase-1 and phase-2 of the study. Individuals with a history of pituitary disease or with PRL ≥35 ng/ml, taking antipsychotic and SSRI anti-depressive agents at phase-1 were excluded in both phases of the study

	Phase-1 (*n* = 2845)		Phase-2 (*n* = 2241)	
	Mean	SD	Mean	SD
Age (years)	60.0	11.0	63.5	10.6
Body mass index (kg/m^2^)	27.8	4.1	27.9	4.3
Waist circumference (cm)	98.3	11.7	99.5	11.8
Prolactin (ng/ml)	8.28	3.94	7.45	3.74
Glucose (mmol/l)	5.65	1.45	5.53	1.43
	%		%	
Smoking (ever)	71.8	--	70.6	--
Smoking (current)	17.1	--	21.4	--
Alcohol consumption (every day)	16.7	--	23.4	--
Diabetes (%)	7.8	--	9.3	--
Cancer (%)	5.5	--	9.6	--
Stroke (%)	3.6	--	5.3	--
Cardiovascular disease (%)	36.9	--	--	--
Vascular disease (%)	--	--	7.5	--
Heart condition (%)	--	--	22.8	--
Angina (%)	--	--	12.8	--
Myocardial infarction (%)	--	--	15.4	--
Congestive heart failure (%)	--	--	16.1	--
Other (%)	--	--	27.0	--
Epilepsy (%)	0.7	--	0.6	--
All-cause mortality	--	--	5.9	--
	**Median**	**IQR**	**Median**	**IQR**
BDI scores	6	2–10	5	2–9
HOMA-IR	2.26	1.45–3.64	2.18	1.48–3.35
Estradiol (pmol/l)	70.1	56.5–86.9	--	--
TT (nmol/l)	16.2	12.6–20.4	15.9	12.5–20.0
FT (pmol/l)	289	239–351	280	227–333
E2/TT	4.4	3.4–5.5	--	--
E2/FT	0.24	0.19–0.31	--	--

BDI, Beck depression inventory; HOMA-IR, Homeostatic model assessment for insulin resistance; TT, total testosterone; FT, free testosterone, E2, estradiol

The prevalences of diabetes (7.8%), cancer (5.5%) and stroke (3.6%) in phase-1 also increased in phase-2 to 9.3%, 9.6%, and 5.3%, respectively, whilst 6.9% developed type 2 diabetes, including those with a new fasting blood glucose ≥ 7 mmol/l (i.e. newly diagnosed) by the time of phase-2 examination. Overall, cardiovascular disease was present in 36.9% phase-1 and individual cardiac conditions were 7.5–22.8% in phase-2. Epilepsy was similarly present in both phases, 0.7% and 0.6% respectively. There were 5.9% of men died following phase-1 of the study. The median BDI scores were 6 in phase-1 and 5 in phase-2. Amongst 209 individuals with diabetes, 186 were considered to have type 2 as they were treated by oral antihyperglycemic agents, including 23 men who were treated with a combination of antihyperglycemic agents and insulin. There were 23 men (0.8% of total sample) treated with insulin alone and were therefore presumed to have type 1 diabetes.

**Fig. 1:**
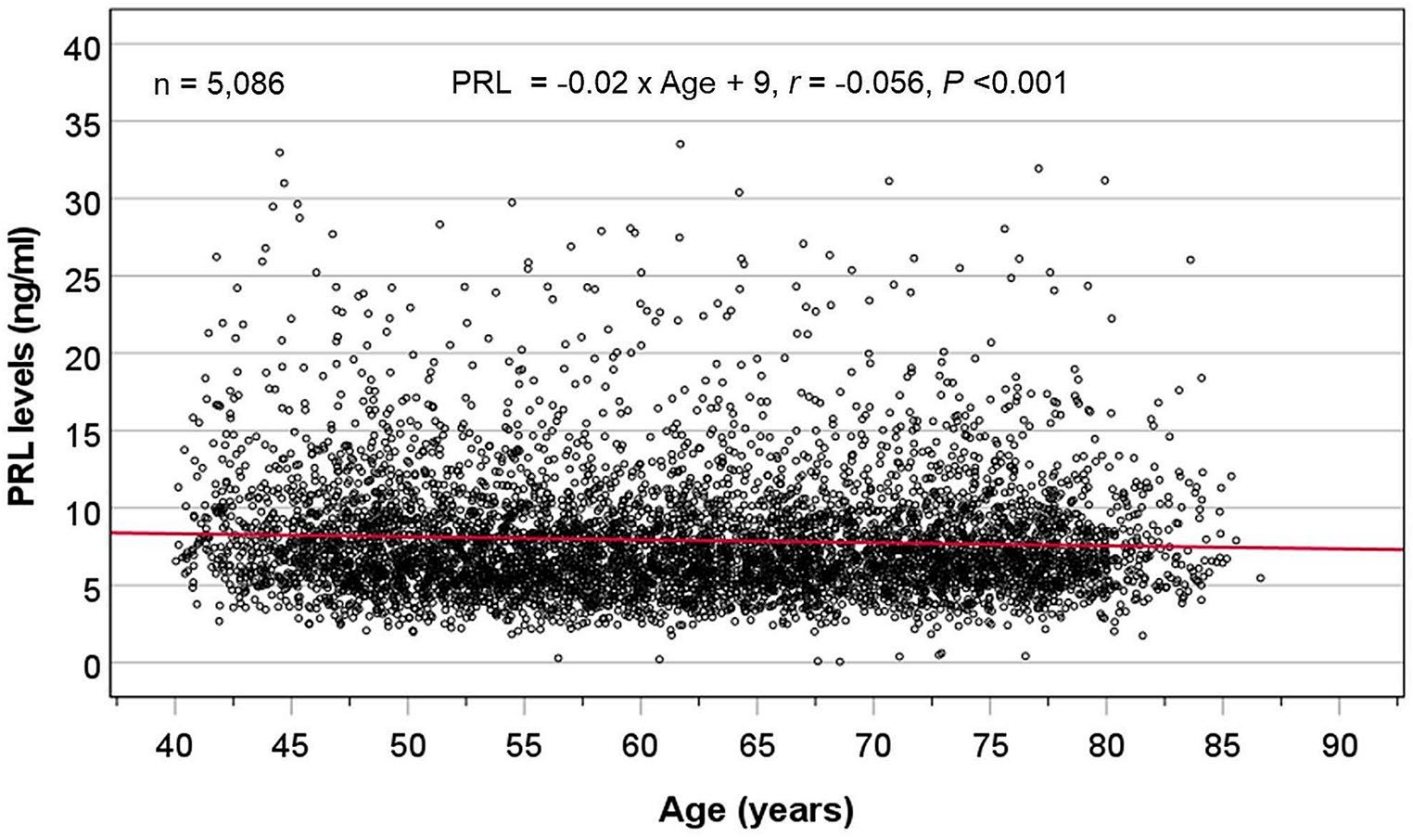
Scatter plots showing PRL levels against age: ranging from 40 to 86 years (up to 80 years in phase-1)

**Fig. 2:**
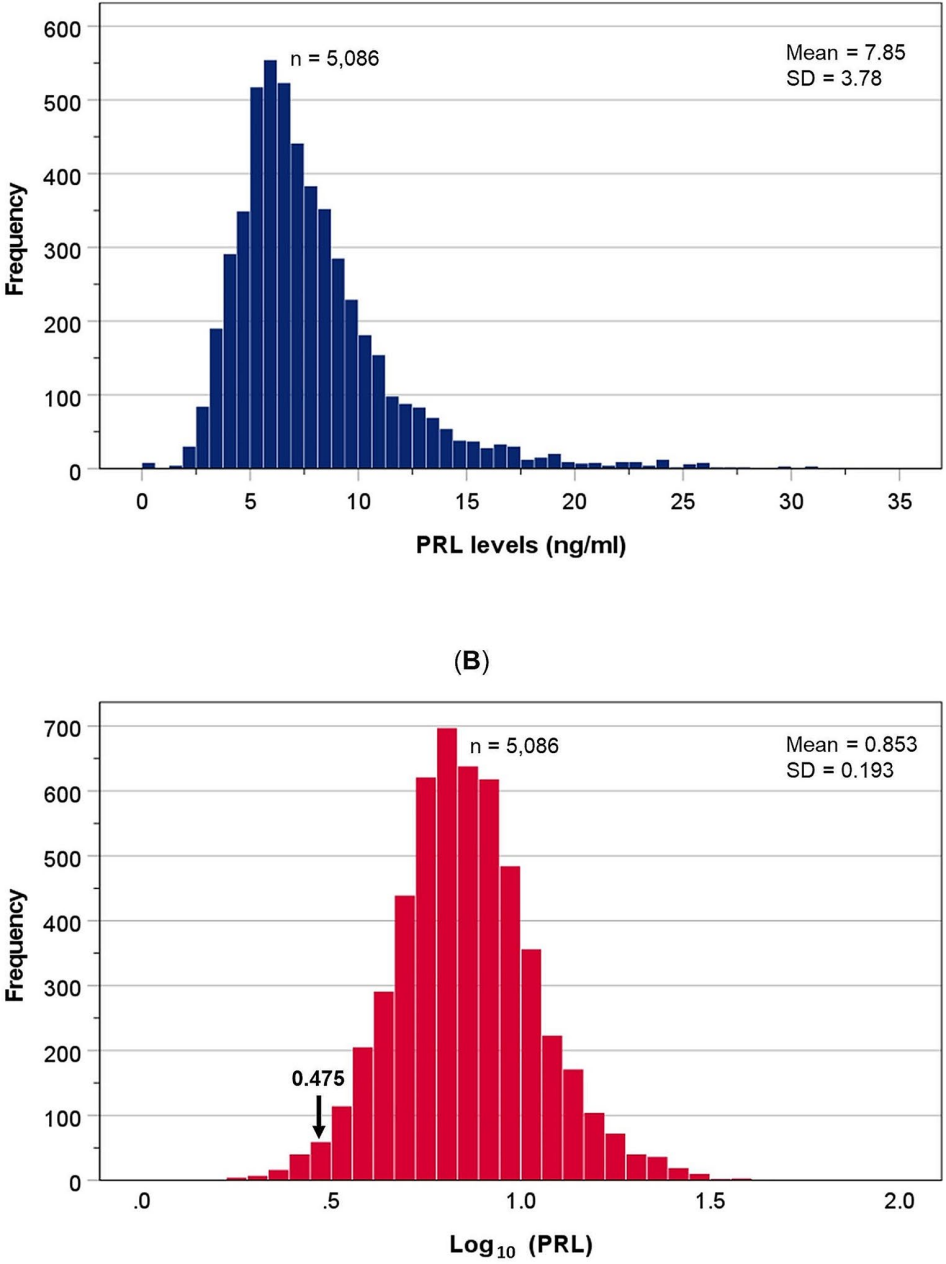
Distribution of PRL in men before (**A**) and after log-transformation. Arrow indicates value correspond to mean? 2SD (0.853? 1.96 []0.139 = 0.475) (**B**).

Scatter plot shows that the levels of PRL minimally declined with age (slope = -0.018) (Fig. 1), and did not correlate with BMI. The distribution of PRL was positively skewed (Fig. 2A) therefore data were log_10_-transformed to a normal distribution (Fig. 2B). The resultant transformed distribution had a mean value of 0.853 and SD of 0.193 (antilog-transformed values of mean = 7.13 ng/ml and SD = 1.56 ng/ml). Based on the two-sigma empirical rule, the threshold at 2.5% of the log_10_ PRL distribution calculated as 0.853 – (1.96[]0.193) = 0.475, which is equivalent to 2.98 ng/ml after conversion by anti-logarithmic transformation.

The levels of PRL did not relate to most clinical conditions considered, including cardiovascular diseases (myocardial infarct, congestive heart failure, vascular disease and stroke), epilepsy, cancer or mortality (Table 2), except for an inverse relationship with diabetes: the prevalences of diabetes were 8.6%, 12.7% and 24.1% in patients within phase-1 PRL groups of 5.0-34.9 ng/ml to 3.0-4.9 ng/ml and 0.3–2.9 ng/ml, respectively. At phase-1, compared to men in the highest PRL category (5-34.9 ng/ml), the adjusted risk of type 2 diabetes was significantly greater in those with lower PRL levels (3-4.9 ng/ml): OR (95%CI) = 1.56 (1.03–2.37), and in those in the lowest PRL category (0.3–2.9 ng/ml): OR = 4.61 (1.82–11.66).

**Table 2:** Rates of conditions and mortality in different categories of PRL

	PRL = 5.0-34.9 ng/ml (*n* = 2427)	PRL = 3.0-4.9 ng/ml (*n*-376)	PRL 0.3–2.9 ng/ml (*n* = 42)	Group differences (*P*)*
	%	%	%	Fisher exact tests
Cancer	9.6	9.6	10.7	0.937
Stroke	5.0	7.4	3.4	0.208
Vascular disease	7.5	6.8	12.5	0.559
Myocardial infarction	15.3	14.5	27.3	0.514
Congestive heart failure	15.4	20.4	10.0	0.379
Epilepsy	0.6	0.7	0	0.741
All cause mortality	5.9	6.4	4.8	0.928
Diabetes	8.6	12.7	24.1	0.002
	**Mean (SD)**	**Mean (SD)**	**Mean (SD)**	**ANOVA**
Body mass index	27.8 (4.3)	28.1 (3.8)	28.7 (3.8)	0.369
Waist circumference	99.3 (12.0)	100.6 (10.3)	102.7 (9.4)	0.083
Fasting glucose (mmol/l)	5.61 (1.34)	5.83 (1.72)	6.40 (3.41)	< 0.001
	**Median (IQR)**	**Median (IQR)**	**Median (IQR)**	**Kruskal-Wallis test**
BDI scores	6 (2–10)	6 (3–10)	7 (3.5–12.5)	0.187
HOMA-IR	2.25 (1.44–3.65)	2.33 (1.52–3.43)	2.71 (1.38–4.23)	0.737
Estradiol (pmol/l)	70.7 (57.2–87.6)	67.2 (54.5–83.9)	62.0 (51.9–76.9)	< 0.001
Total testosterone (nmol/l)	16.3 (12.8–20.4)	15.7 (12.1–20.2)	16.0 (12.4–20.4)	0.213
Free testosterone (pmol/l)	291 (241–352)	276 (224–336)	285 (227–337)	0.002
E2/TT	4.37 (3.43–5.58)	4.24 (3.42–5.21)	3.96 (3.29–4.76)	0.098
E2/FT	0.24 (0.19–0.31)	0.24 (0.20–0.30)	0.23 (0.19–0.30)	0.663

BDI, Beck depression inventory; HOMA-IR, Homeostatic model assessment for insulin resistance; TT, total testosterone; FT, free testosterone, E2, estradiol

**Fig. 3:**
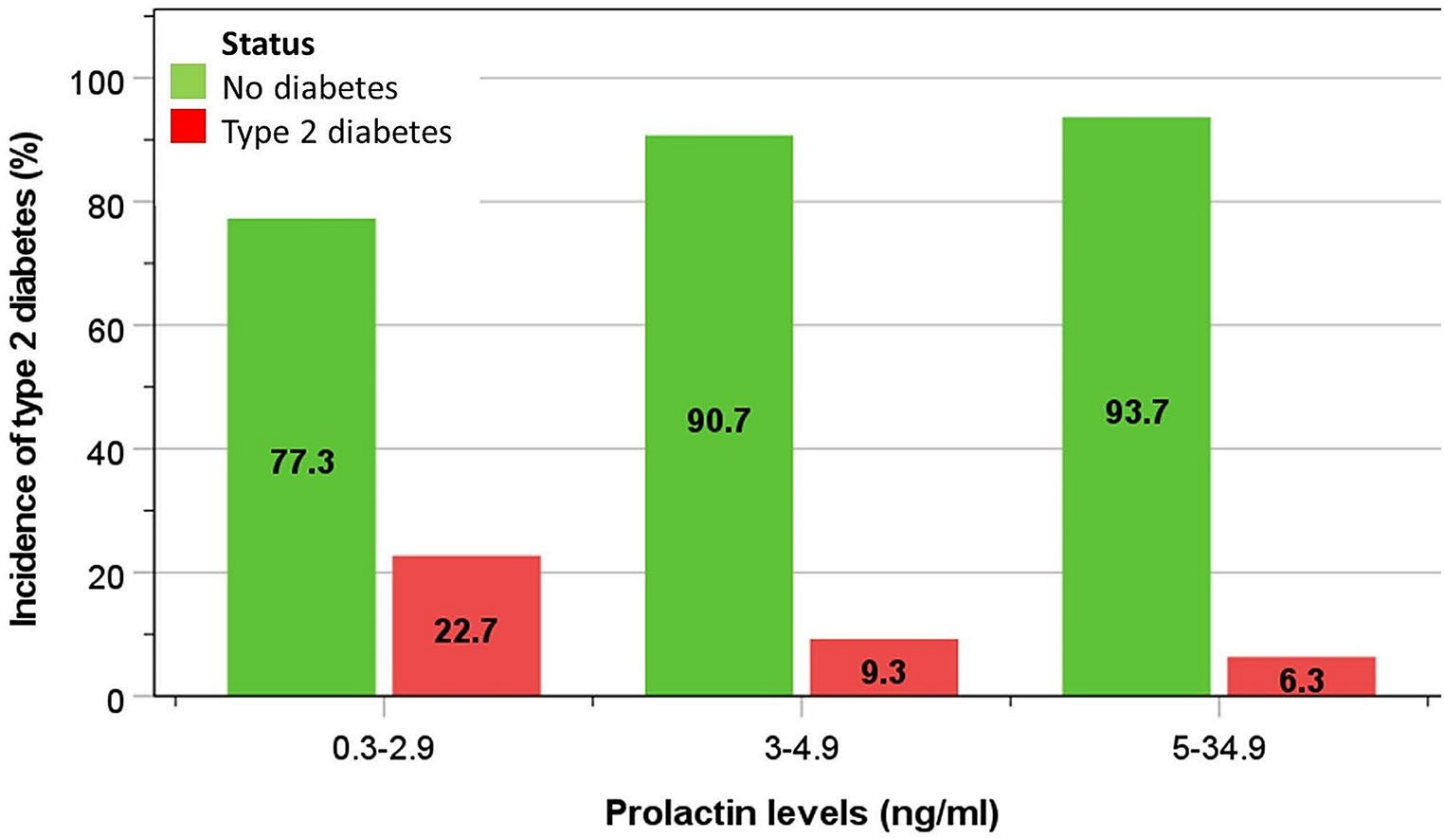
Incidence of type 2 diabetes developed by the time of phase-2 study according to phase-1 PRL levels

**Fig. 4:**
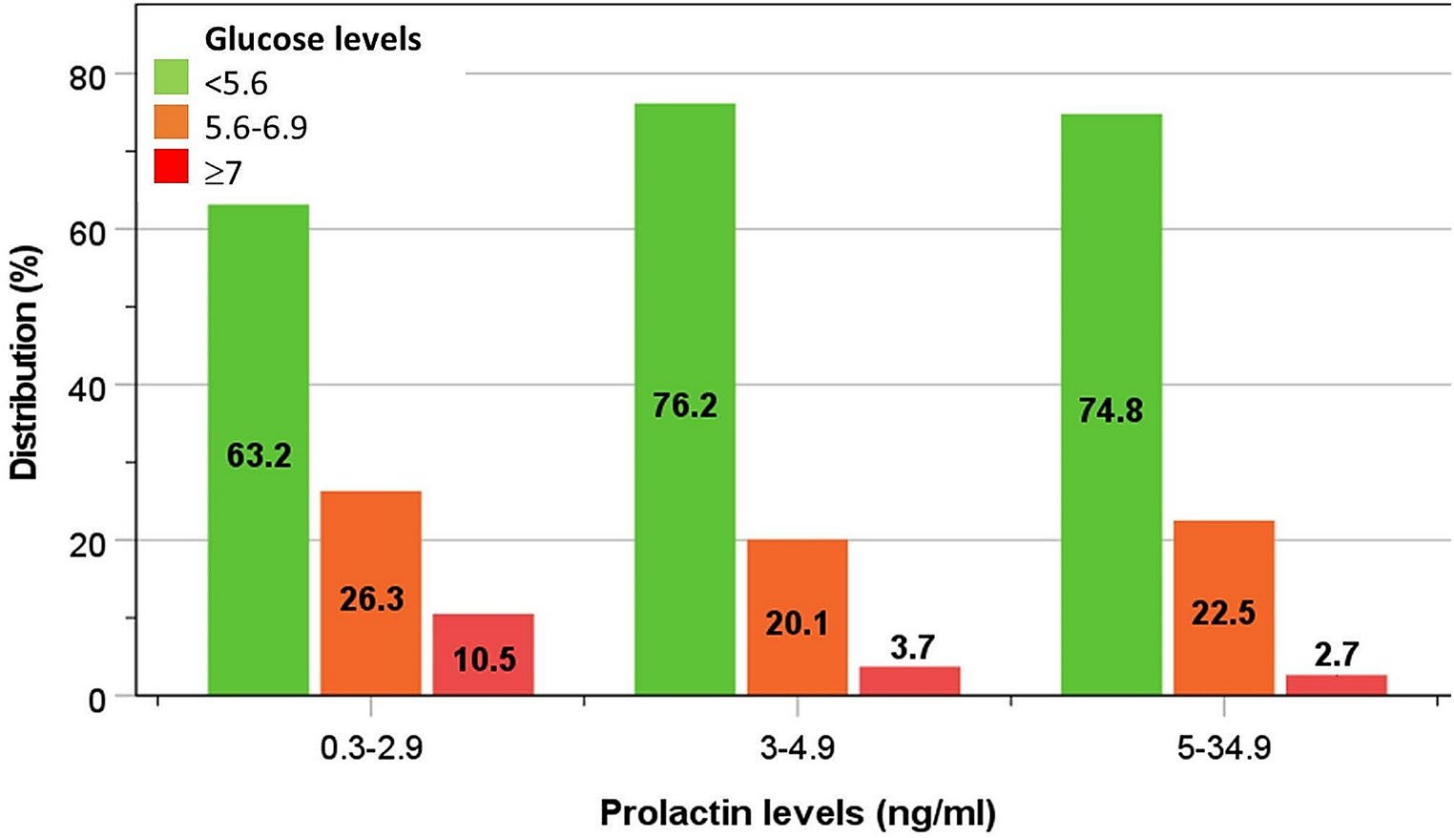
Distribution of fasting blood glucose levels according to different categories of phase-1 PRL levels amongst men without a previous history of diabetes

The incidences of type 2 diabetes at phase-2 examination in men with phase-1 PRL levels of 5-34.9 ng/ml (considered the normal range), 3-4.9 ng/ml, and 0.3–2.9 ng/ml were 6.3%, 9.3% and 22.7%, respectively (Fig. 3). Compared to men in the highest PRL category (5-34.9 ng/ml), the adjusted risk of type 2 diabetes increased progressively in those with lower PRL levels (3-4.9 ng/ml): OR (95%CI) = 1.59 (0.93–2.71), and in those in the lowest PRL category (0.3–2.9 ng/ml): OR = 5.45 (1.78–16.62) (Table 3). An increasing trend in prediabetes and diabetes based on fasting blood glucose levels was observed with lower categories of PRL (Fig. 4).

**Table 3:** Risk of development of diabetes at phase-2 amongst men with lower PRL levels at phase-1

	PRL = 5.0-34.9	PRL = 3.0-4.9			PRL 0.3–2.9		
Unadjusted	(Reference)	OR	95% CI	*P*	OR	95% CI	*P*
Type 2 diabetes	1	1.50	0.92–2.47	0.106	4.30	1.57–12.02	0.005
**Adjusted for age, study centres, smoking, alcohol consumption and body mass index**							
Type 2 diabetes	1	1.59	0.93–2.71	0.092	5.45	1.78–16.62	0.003
**Adjusted for age, study centres, smoking, alcohol consumption and waist circumference**							
Type 2 diabetes	1	1.63	0.95–2.80	0.074	5.48	1.78–16.90	0.003

There were no associations of PRL with HOMA-IR or TT. Compared to the highest PRL category (5-34.9 ng/ml), those with PRL in lower categories of PRL had lower levels of E2 and FT, but there were no differences in E2/TT or E2/FT ratio (Table 2).

## Discussion

In this study of a large sample of European men of wide age range and free of acute illnesses, PRL levels did not vary substantially with age or adiposity, and 2.5% of men had PRL levels < 3 ng/ml (< 64 mIU/l). We therefore propose that a PRL of 3 ng/ml indicates the lower limit of normal serum PRL. Below this threshold, considered to be biochemical hypoprolactinemia, a substantially increased risk of developing type 2 diabetes and prediabetes was observed.

The present study used the two-sigma empirical rule to determine the group of individuals with a PRL level within the lowest 2.5% amongst all participants, i.e. 95% of the participants had PRL within two SD about the mean, whilst the remaining 2.5% were above the upper limit threshold. This technique is widely used to determine the limits of reference for clinical measurements [29, 30], educational attainments [31, 32], as well as its application in business models [33], and in the fields of physics [25, 34] and engineering [35].

The levels of PRL may be affected by variation in biological factors. Although the number of lactotrophs appears to be similar in both sexes and does not change with age [36], a study of individuals aged 15–65 years showed increasing age was related to decreasing prolactin levels in women but increasing levels in men [7], evidently due to decreased levels of estrogens in the former and increased estrogen/androgen proportion in the latter. By contrast, our study of men aged 40–86 years showed a minimal decrease in PRL levels with age. A previous study of PRL levels sampled over 24-hours showed the mean PRL concentration was higher in women and in individuals with higher BMI, whilst the peak PRL concentration was negatively associated with age [8]. Our study in men has shown that PRL levels did not relate to BMI. Furthermore, the same population of participants were sampled 3 to 5.7 years later showing no substantial changes in PRL levels. Thus, the threshold described herein for defining hypoprolactinemia is applicable to men aged 40–86, independent of adiposity.

Circulating PRL plays an important role in health, with pleiotropic biological actions including breast development, lactation, and maternal behaviours (nursing and food intake), as well as metabolic homeostasis such as regulation of body weight and adipose tissue, bone, skin and hair follicles, pancreatic β-cells, and adrenal response to stress [10, 37, 38]. The paucity of research on hypoprolactinemia is therefore surprising [39]. Cross-sectional studies have shown an association between low levels of PRL and the development of metabolic syndrome (including type 2 diabetes) and sexual dysfunction in middle-aged and older men [40], including one study from EMAS [41]. Findings from phase-1 of the association between low PRL and increased rates of diabetes are therefore consistent with reports from previous cross-sectional studies. The present results further demonstrate an increased risk of development (incidence) of type 2 diabetes, as well as prediabetes, at even lower threshold of PRL (3 ng/ml). An up-to-date review of this topic has been conducted (see accompanying paper in this issue) [42]. The underlying mechanisms linking hypoprolactinemia and development of type 2 diabetes are unclear, but the association of PRL with diabetes is independent of age, adiposity, smoking, and alcohol consumption. A potential explanation is the lower T, and consequently lower E2, in diabetic men; E2 is well-established stimulating factor of PRL secretion [43]. Interestingly, at physiological level in men, as demonstrated in our study, E2 in parallel with FT, were significantly reduced in those with lower PRL levels. However, PRL did not relate to E2/T (an indicator of aromatisation). Studies have shown that pharmacological doses of estrogen (1.5 mg estradiol benzoate once a day for nine days), in conjunction with gonadotrophin releasing hormone, given to castrated men led to a significant rise in PRL [44].

Studies are warranted in those with hypoprolactinemia to see if raising their PRL levels, e.g. with a dopamine antagonist, would improve glycaemia or insulin sensitivity. Our findings are in contrast to those observed in previous studies where higher PRL levels were found to associate with greater risk of incident diabetes [45], and treatment to reduce PRL levels with a dopamine agonist (Bromocriptine) improves glycaemic control.

In this study of middle-aged and older men, including those with hypoprolactinemia, we observed no associations between circulating PRL levels with other conditions including cancer, cardiovascular disease, depressive symptoms, or mortality. Previous studies focussed solely on the associations of higher levels of PRL with health outcomes, and observed an association of higher PRL levels with increased risk of breast cancer in post-menopausal women [46], including those who used hormone replacement therapy [47]. Other studies have observed an increase in cardiovascular and all-cause mortality in men and women, and cancer death in men, with higher levels of PRL [48].

Hypoprolactinemia itself is asymptomatic in men. It may be induced by certain conditions which directly cause a destruction to the anterior pituitary such Sheehan’s syndrome, brain tumours causing mass effect (e.g. craniopharyngiomas), hypophysectomy, dopamine agonists (e.g. cabergoline, bromocriptine, quinagolide and pergolide), infections (e.g. tuberculosis and histoplasmosis), hypophysitis, and infiltrative disease processes such as sarcoidosis and hemochromatosis. Other chronic conditions including autoimmune diseases such as systemic lupus erythematosus, antiphospholipid syndrome, rheumatoid arthritis, multiple sclerosis, systemic sclerosis, autoimmune thyroid disease, and coeliac disease may also reduce prolactin production [10, 11]. Other rare causes of hypoprolactinemia include abnormal development of lactotrophs due to genetic defects (*POU1F1, PROP1, LHX3, LHX4, HESX1, OTX2* and *IGSF1* loss-of-function mutations) [10] (Table 4). The present study did not specifically exclude these conditions, or related medical treatments.

**Table 4:** Causes of hypoprolactinemia

**Lesions or destruction of pituitary tissue**
Sheehan’s syndrome
Brain tumours causing mass effect (craniopharyngiomas)
Hypophysitis
Autoimmune diseases (systemic lupus erythematosus, antiphospholipid syndrome, rheumatoid arthritis, multiple sclerosis, systemic sclerosis, autoimmune thyroid disease, and coeliac disease)
Traumatic brain injury
Surgery including hypophysectomy
Cranial radiotherapy
Infections (tuberculosis and histoplasmosis)
**Drugs**
Dopamine agonists (cabergoline, bromocriptine, quinagolide and pergolide)
**Abnormal lactotroph cell development (genetic causes)**
*POU1F1*, *PROP1*, *LHX3*, *LHX4*, *HESX1*, *OTX2* and *IGSF1* loss-of-function mutations
**Idiopathic hypoprolactinemia**

This study has some limitations. Although the population assessed are only in males aged 40–86 year, this has the advantage of not including young ages or women, in whom large PRL variations occur due to hormonal changes, for example estrogen levels, both from endogenous and exogenous (contraceptive pill) sources. The cut-off value at 5 ng/ml is arbitrary but evidence from previous EMAS analysis [41] showed an association of specific sexual and psychological characteristics and unhealthy metabolic phenotype men with PRL below this threshold. The strength of the study lies in its very large population of community-dwelling men; there were no hospitalised patients. The follow-up study (phase-2) enabled us to predict the development of type 2 diabetes, which is a unique feature of this study. The other advantage is that PRL values were all measured with a single assay, and remained very stable within individuals: there were no substantial age-related changes across age groups from 40 to 86 years, and PRL values obtained from the same individuals did not alter over a period of 3 to 5.7 years. We recognise that the primary goal of EMAS was not to find or estimate the lower limit of serum PRL level. Thus, since all possible pathologic conditions other than type 2 diabetes mellitus associated with hypoprolactinemia were not extensively investigated. Further research on the development of other diseases in patients with hypoprolactinemia is suggested.

In conclusion, we have for first time proposed a lower reference value for PRL of 3 ng/ml (64 mlU/l), with lower levels indicating clinical hypoprolactinemia and a strong association with type 2 diabetes. The weaker association of PRL < 5 ng/ml (106 mIU/l) with the presence of diabetes strengthens this evidence and merits further study.

## Data Availability

No datasets were generated or analysed during the current study.
